# Supplementary material for: A Comprehensive Assessment of Ultraviolet-Radiation-Induced Mutations in Flammulina filiformis Using Whole-Genome Resequencing
Source: J Fungi (Basel). 2024 Mar 20;10(3):228. doi: 10.3390/jof10030228 (PMC10971301; doi:10.3390/jof10030228)
Supplement: Supplementary file 1 [file jof-10-00228-s001.zip › Supplementary Material S8/KEGG annotation/out/64381550635650.os/KO/out_map/map04020.html]

KEGG PATHWAY: Calcium signaling pathway - Reference pathway


|  |  |
| --- | --- |
| **Calcium signaling pathway - Reference pathway** |  |

[
Pathway menu
| Organism menu
| Pathway entry
| Show description
| User data mapping
]

Ca2+ that enters the cell from the outside is a principal source of signal Ca2+. Entry of Ca2+ is driven by the presence of a large electrochemical gradient across the plasma membrane. Cells use this external source of signal Ca2+ by activating various entry channels with widely different properties. The voltage-operated channels (VOCs) are found in excitable cells and generate the rapid Ca2+ fluxes that control fast cellular processes. There are many other Ca2+-entry channels, such as the receptor-operated channels (ROCs), for example the NMDA (N-methyl-D-aspartate) receptors (NMDARs) that respond to glutamate. There also are second-messenger-operated channels (SMOCs) and store-operated channels (SOCs).
The other principal source of Ca2+ for signalling is the internal stores that are located primarily in the endoplasmic/sarcoplasmic reticulum (ER/SR), in which inositol-1,4,5-trisphosphate receptors (IP3Rs) or ryanodine receptors (RYRs) regulate the release of Ca2+. The principal activator of these channels is Ca2+ itself and this process of Ca2+-induced Ca2+ release is central to the mechanism of Ca2+ signalling. Various second messengers or modulators also control the release of Ca2+. IP3, which is generated by pathways using different isoforms of phospholipase C (PLCbeta, delta, epsilon, gamma and zeta), regulates the IP3Rs. Cyclic ADP-ribose (cADPR) releases Ca2+ via RYRs. Nicotinic acid adenine dinucleotide phosphate (NAADP) may activate a distinct Ca2+ release mechanism on separate acidic Ca2+ stores. Ca2+ release via the NAADP-sensitive mechanism may also feedback onto either RYRs or IP3Rs. cADPR and NAADP are generated by CD38. This enzyme might be sensitive to the cellular metabolism, as ATP and NADH inhibit it.
The influx of Ca2+ from the environment or release from internal stores causes a very rapid and dramatic increase in cytoplasmic calcium concentration, which has been widely exploited for signal transduction. Some proteins, such as troponin C (TnC) involved in muscle contraction, directly bind to and sense Ca2+. However, in other cases Ca2+ is sensed through intermediate calcium sensors such as calmodulin (CALM).

|  |  |
| --- | --- |
| Reference pathway | 100% |
